# Evaluation of clinical outcome measures for HAM/TSP

**DOI:** 10.1186/1742-4690-12-S1-P67

**Published:** 2015-08-28

**Authors:** Takayuki Kikuchi, Aika Sawabe, Yui Negishi, Airi Noda, Yuji Hirai, Eisuke Inoue, Miyuna Kimura, Kentaro Sato, Natsumi Araya, Naoko Yagishita, Tomoo Sato, Yoshihisa Yamano

**Affiliations:** 1 St. Marianna University School of Medicine, Kawasaki, Japan; 2 Division of Statistical Analysis, National Center for Child Health and Development, Tokyo, Japan; 3 Institute of Medical Science, St. Marianna University School of Medicine, Kawasaki, Japan

## 

HTLV-1-associated myelopathy/tropical spastic paraparesis (HAM/TSP) is a rare disease for which there has been no effective therapy. Therefore, there is an urgent need to develop new effective drugs. To promote new drug development, we first need to identify appropriate efficacy outcome measures and then conduct clinical trials for the treatment of HAM/TSP. In this study, we aimed to determine what outcome measures are more suitable for primary outcome measures in clinical trials for HAM/TSP patients. First, we compared some existing outcome measures such as 10-meter walk test (10mWT), Osame's motor disability score (OMDS) and Expanded Disability Status Scale (EDSS) in terms of clinical relevance, objectivity, quantifiability and so on. Second, we measured 10mWT, 2-minute walk test (2minWT), OMDS, HAQ-disability index (HAQ-DI) in 60 patients with HAM/TSP and analyzed the correlation between them. Last, we compared 10mWT with 2minWT in terms of sample size required for a clinical trial. As a result of a first comparison with some existing outcome measures, we determined that outcome measures of gait disturbance were appropriate for primary outcome measures in clinical trials because gait disturbance is most common symptoms of HAM/TSP. Among the outcome measures of gait disturbance, we selected 10mWT and 2minWT based on objectivity and quantifiability. Both 10mWT and 2minWT correlated well with OMDS (rs = 0.702 and 0.708). Likewise, both outcome measures also correlated well with HAQ-DI. Furthermore, estimated sample size required to detect a 20% improvement of 10mWT was 26 cases that was the same as that of 2minWT. In conclusion, this study suggested that both 10mWT and 2minWT are useful as primary outcome measures in clinical trials for HAM/TSP patients.

